# Biomimetic Collagen/Zn^2+^-Substituted Calcium Phosphate Composite Coatings on Titanium Substrates as Prospective Bioactive Layer for Implants: A Comparative Study Spin Coating vs. MAPLE

**DOI:** 10.3390/nano9050692

**Published:** 2019-05-03

**Authors:** Ionela Andreea Neacsu, Laura Vasilica Arsenie, Roxana Trusca, Ioana Lavinia Ardelean, Natalia Mihailescu, Ion Nicolae Mihailescu, Carmen Ristoscu, Coralia Bleotu, Anton Ficai, Ecaterina Andronescu

**Affiliations:** 1 Faculty of Applied Chemistry and Materials Science, University Politehnica of Bucharest, Polizu Street No.1, 011061 Bucharest, Romania; neacsu.a.ionela@gmail.com (I.A.N.); arsenielaura@yahoo.com (L.V.A.); roxanatrusca@yahoo.com (R.T.); dy4_ioana@yahoo.com (I.L.A.); anton_ficai81@yahoo.com (A.F.); 2 National Institute for Laser, Plasma and Radiation Physics, Atomiştilor Street No. 409, 077125 Măgurele, Romania; natalia.serban@gmail.com (N.M.); ion.mihailescu@inflpr.ro (I.N.M.); carmen.ristoscu@inflpr.ro (C.R.); 3 Stefan S. Nicolau’ Institute of Virology, Romanian Academy, 011061 Bucharest, Romania; cbleotu@yahoo.com; 4 Academy of Romanian Scientists, Splaiul Independentei Street No. 54, 011061 Bucharest, Romania

**Keywords:** Zn^2+^ substituted Coll-CaPs biomimetic layers, MAPLE, spin coating

## Abstract

Synthesis of biomimetic materials for implants and prostheses is a hot topic in nanobiotechnology strategies. Today the major approach of orthopaedic implants in hard tissue engineering is represented by titanium implants. A comparative study of hybrid thin coatings deposition was performed by spin coating and matrix-assisted pulsed laser evaporation (MAPLE) onto titanium substrates. The Collagen-calcium phosphate (Coll-CaPs) combination was selected as the best option to mimic natural bone tissue. To accelerate the mineralization process, Zn^2+^ ions were inserted by substitution in CaPs. A superior thin film homogeneity was assessed by MAPLE, as shown by scanning electron microscopy (SEM) and Fourier transform infrared (FTIR) microscopy. A decrease of P-O and amide absorbance bands was observed as a consequence of different Zn^2+^ amounts. A variety of structural modifications of the apatite layer are then generated, which influenced the confinement process towards the collagen template. The in-vitro Simulated Body Fluid (SBF) assay demonstrated the ability of Coll/Zn^2+^-CaPs coatings to stimulate the mineralization process as a result of synergic effects in the collagen-Zn^2+^ substituted apatite. For both deposition methods, the formation of droplets associated to the growth of CaPs particulates inside the collagen matrix was visualized. This supports the prospective behavior of MAPLE biomimetic coatings to induce mineralization, as an essential step of fast implant integration with vivid tissues.

## 1. Introduction

The prevention of post-surgical inflammation or rejection after the implantation of metallic devices is of growing interest in tissue engineering. In particular, Ti prostheses exhibit a pronounced tendency of corrosion, releasing abrasive metallic particles (as a result of redox processes) into the physiological medium [1,2]. Then, the corrosion leads to undesirable consequences, such as inflammation and infections that inhibit the artificial bone reconstruction. Another inconvenience in using common Ti orthopaedic implants resides in the low capacity to promote the interaction between the natural tissue and device [3]. The most important objective to overcome these disadvantages is therefore oriented towards the fabrication of smart biomaterials with enhanced biocompatibility, osseointegration, high surface properties and superior chemical stability.

An intelligent solution to address these limitations resorts to the deposition of thin coatings on Ti substrates. The aim of our work reflects a comparative study of two deposition methods—spin coating and matrix-assisted pulsed laser evaporation (MAPLE)—involved in the production of biocompatible collagen-Zn^2+^ substituted calcium phosphate thin coatings.

The materials developed are able to display a biomimetic behavior similar to the bone tissue. Materials selection was inspired by the composition of mammalian bone, which consists of a mixture of proteins and minerals [4]. The main inorganic component is hydroxyapatite (HAp) and is in a proportion of ~50 wt% and ~70 vol%, depending on sex and age. The rest is water and matrix and is pre-existing serving as a scaffold for mineralization. Collagen, the most abundant protein in the body, reaches about 90 wt% in bone matrix. Composite materials based on collagen and calcium phosphates (Coll-CaPs) are usually considered a good choice for hard tissue engineering because of the structural and compositional similarity with the natural bone. The most promising systems are therefore bone-like apatite layers designed by calcium phosphates (CaP) particles (including hydroxyapatite-HAp and tricalcium phosphate-TCP as major components) embedded in a collagen template [5,6,7]. Loading the biomimetic organic-inorganic matrix with platelet-rich plasma (PRP), bone morphogenetic proteins (BMPs) or bisphosphonates facilitates the osseous regeneration [8,9,10]. Moreover, complementary functional motifs dependent on supramolecular interactions (e.g., hydrogen bonds, electrostatic interactions, etc.) define the advantageous selection of these two components [7,11]. An advanced biocompatibility could be envisaged by using HAp, which in association with TCP improves the properties of the final implant in respect with the mechanical characteristics and bioactivity [12,13]. The apatite network enables the cationic exchanges via substitution processes, without altering the final features of the implant. Some studies highlighted the substantial benefits of using Ag^+^ and Zn^2+^ cations as substitution agents in preventing the post-infection due to the enhanced antimicrobial activity [14,15,16,17]. Ag^+^ is susceptible to be more toxic than Zn^2+^ even at ppm level concentrations, the ionic charge imbalance between Ca^2+^ and Ag^+^ cations leading to structural modifications of the calcium-phosphate network [18]. On the other side, Zn^2+^ ions exhibit not only advanced antimicrobial behavior, but also promote the osteointegration processes [19].

Besides the importance of organic and inorganic synthons involved in the formation of the coating, the deposition method plays a major role in obtaining the desired chemical and biological properties. Requirements such as biocompatibility, osteoregeneration, or resorbability are generally related to an improved interaction of the implant with the natural tissue and physiological medium.

Generally, the coatings obtained via spin coating are not uniformly distributed on the metallic substrate, the random distribution of the composite generating differences in the properties of the implant, minimizing a complete regeneration (e.g., an imbalanced mineralization process in some areas that are characterized by an excess of composite material) and also areas strongly exposed to corrosion and inflammatory pitting points [20]. Nevertheless, the spin coating method could be properly balanced by easily monitoring some parameters (e.g., time and rotation speed, acceleration and deceleration), combined with chemical modifications of the deposited organic material (e.g., cross-linking with imines or aldehydes to preserve the collagen structure) or inorganic precursors. Also, the modifications of the metallic substrate are developed prior to the deposition method (e.g., chemical etching to offer an advanced adherence of the biocomposite to the disk). In contrast to spin coating, the matrix-assisted pulsed laser evaporation (MAPLE) technique promises a significant advantage by preserving the collagen/CaP ratio, due to the prevention of collagen denaturation during deposition process [21,22,23]. One therefore expects significantly improvement of both collagen-apatite interaction, as well as adherence to the Ti substrate. In MAPLE, the uniformity of the coating is easily achieved due to the additive character of the process [24]. More exactly, one can stop the deposition as soon as the desired thickness and uniformity are reached.

One should stress that, in contrast to other well-recognized deposition methods, like plasma (magnetron sputtering, electron/ion bombardment, pulsed laser deposition, laser induced forward transfer) or other techniques (Molecular Beam Epitaxy, Atomic Layer Deposition), both spin coating and MAPLE are conforming to requirements of safe transfer of organic materials. Indeed, they either do not use high temperature processing or protect via ice or other shields the delicate compounds like collagen against irreversible damage by temperature and/or laser radiation. We consider therefore that a comparison between the relative performances of the two techniques is relevant for potential bioactive layer deposition on implants.

Spin Coating and MAPLE were comparatively applied in the present study for thin films deposition on chemically etched Ti substrates. The Coll/CaPs biocomposite coatings with different Zn^2+^ contents (within 0–2 wt%) were investigated concerning morphological and structural characteristics by infrared mapping (IR), scanning electron microscopy (SEM), Fourier transform infrared spectroscopy (FTIR), energy dispersive spectrometry (EDAX), but also in correlation with their in-vitro functional properties in terms of an artificial mineralization process in simulated body fluid (SBF).

## 2. Materials and Methods

### 2.1. Materials

Calcium nitrate tetrahydrate (Ca(NO_3_)_2_·4H_2_O), zinc nitrate hexahydrate(Zn(NO_3_)_2_·6H_2_O), ammonium hydroxide (NH_3_ aq.) (30 wt% solution), hydrofluoric acid (HF), hydrogen peroxide (H_2_O_2_) (30 wt% solution), glutaraldehyde (C_5_H_8_O_2_) (25 wt% solution), sodium chloride (NaCl), potassium chloride (KCl), potassium phosphate dibasic trihydrate (K_2_HPO_4_·3H_2_O), magnesium chloride hexahydrate (MgCl_2_·6H_2_O), calcium chloride (CaCl_2_), sodium sulphate (Na_2_SO_4_) and triethanolamine (C_6_H_15_NO_3_) were purchased from Sigma-Aldrich, Darmstadt, Germany. Sodium phosphate dibasic dihydrate (Na_2_HPO_4_·2H_2_O) and nitric acid (HNO_3_) were supplied by Fluka, Riedel-de-Haen, Germany. Sodium bicarbonate (NaHCO_3_) was obtained from Riedel-de-Haen, Seelze, Germany. The collagen hydrogel (1.46 wt% anhydrous substance, pH = 2.5) was supplied by Sanimed International Impex SRL, Bucharest, Romania. HeLa cell culture was provided by American Tissue Culture Collection, Manassas, VA, USA, while Dulbecco’s Modified Eagle’s Medium and bovine serum were obtained from Sigma-Aldrich, St. Louis, MO, USA. Ti substrates (disk-shaped; 12 mm diameter, 0.5 mm thick) were provided by NextMaterials SRL, Milano, Italy [25].

### 2.2. Coll/Zn^2+^-CaPs Hydrogels: Preparation

Coll/Zn^2+^-CaPs hydrogels were synthesized by the in-situ generation of Zn^2+^-substituted calcium phosphates in the collagen gel, the ratio between the collagen and CaPs was set at 1:1 (wt:wt). A Ca^2+^-Zn^2+^ suspension was added dropwise in a flask containing 10 g of collagen hydrogel, kept on an ice bath. The mixture was mechanically stirred for 1 h at 500 rpm. Next, a sodium phosphate dibasic solution was added, the final mixture being stirred for 3 h at 500 rpm until complete homogenization. The co-existence of HAp and TCP as major components in the prepared coatings was further abbreviated as CaPs.

### 2.3. Deposition Methods

#### 2.3.1. Spin Coating

The hydrogels prepared were deposited by spin coating on Ti substrates, with a Laurell WS-650 apparatus (3000 rpm, deposition time = 2 s/per layer, North Wales, PA, USA). Prior to deposition, Ti substrates were mechanically polished and modified by chemical etching in order to assure a suitable roughness benefic for an advanced adherence of the composite. The etching was performed by consecutively immersing the Ti substrates in HF-HNO_3_ solution (ratio = 1:3), and in an H_2_O_2_ 15 wt% solution, respectively. After deposition, the Coll/Zn^2+^-CaPs biocomposite coatings were cross-linked with glutaraldehyde 2.5 wt% solution and maintained at 4 °C for 24 h.

#### 2.3.2. Matrix-Assisted Pulsed Laser Evaporation (MAPLE)

**Figure 1:**
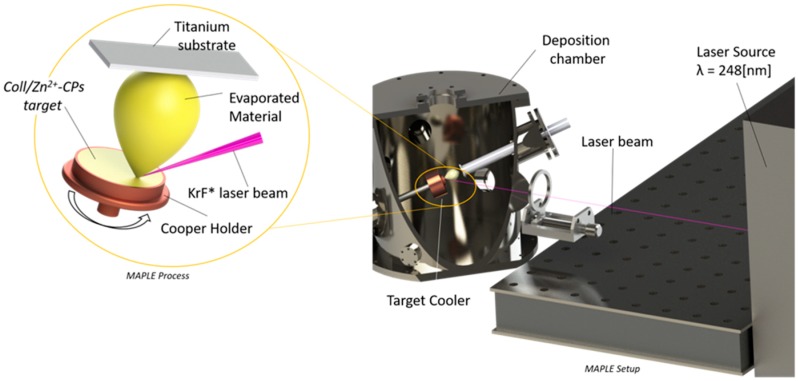
Schematic of matrix-assisted pulsed laser evaporation (MAPLE) set-up.

10 mL of Coll/CaPs hydrogel with different Zn^2+^ contents (0.5, 1 and 2 wt%) of Zn^2+^ have been used for the preparation of one MAPLE target. Before deposition, the target was frozen in a special copper holder at 77 K in liquid nitrogen and maintained at this temperature during deposition, using a cryogenic rotating setting (Figure 1) [26].

The chemically etched Ti substrates were successively cleaned in an ultrasonic bath for 15 min in acetone, alcohol and deionized H_2_O and blown dry with high purity nitrogen before use as substrates for deposition. The experiments were conducted using a COMPEX Pro 205 KrF * (λ = 248 nm, $\tau_{FWHM}\leq$ 25 ns) excimer laser source (Coherent) which was operated at a fluence of 0.5 J/cm^−2^ at a repetition rate of 10 Hz. The laser beam was focused with MgF_2_ cylindrical lens at an angle of 45° on the surface of the cryogenic target. Ti substrate was placed inside the reaction chamber parallel to the Coll/Zn^2+^-CaPs target at a separation distance of 5 cm. For the growth of one film, 50,000 subsequent laser pulses have been applied. In order to obtain a uniform layer and to avoid drilling, the target and substrate were continuously rotated at 50 rpm, while the background pressure inside the deposition chamber was of 2 × 10^−2^ mbar.

### 2.4. Structural and Morphological Analyses

IR mapping analysis was applied to study the homogeneity of Coll/Zn^2+^-CaPs films in correlation with different changes after Zn^2+^ substitutions in the hybrid coating. The analysis was done with an IR Thermo Scientific Nicolet iN10MX microscope (Waltham, MA USA) in reflection mode, at a spectral resolution of 4 cm^−1^ and a spatial resolution of 100 μm × 100 μm. Each spectrum cumulates the co-adding of 16 scans. For higher resolution a cooled MCT detector was used. FTIR spectra were acquired with a spectrophotometer Nicolet iS50R equipped with 3 beam splitters in the (12,500–50) cm^−1^ range. Spectra between 3800–500 cm^−1^ were recorded by co-adding 64 scans at a spectral resolution of 4 cm^−1^. The morphology of the biocomposite coatings was studied via scanning electron microscopy (SEM), with a Quanta Inspect F50 microscope coupled with an energy dispersive spectrometer (EDAX) (Oregon, OR, USA).

### 2.5. In-Vitro Testing in Simulated Body Fluids (SBF)

The in-vitro mineralization process was assessed by the immersion of Coll/Zn^2+^-CaPs composite coatings in SBF (10 mL of SBF/per disk) at 37 °C in a thermostatic bath. After 14 days, the coated Ti disks were characterized by FTIR and SEM analysis. 1 L of SBF solution was prepared according to Kokubo methodology [27]. The concentrations of the ionic species involved in SBF preparation are listed in Table 1. The pH of the final buffer solution is 7.25.

### 2.6. Cell Viability

The cell viability assay was effectuated on simple and SBF immersed Coll/Zn^2+^-CaPs composited deposited via MAPLE technology. The eukaryotic HeLa cell culture was maintained in Dulbecco’s Modified Eagle’s Medium supplemented with 10 wt% heat-inactivated fetal bovine serum at 37 °C in a 5 wt% CO_2_ humid atmosphere. Coll/Zn^2+^-CaPs were plated in 24-well plates and 1 × 10^5^ cells were added onto each MAPLE sample. After 24 h, the Coll/Zn^2+^-CaPs materials were moved to other wells, fixed in 70 wt% ethanol, stained with 50 μg/mL propidium iodide (PI) and washed with phosphate buffer solution (PBS). The Observer D1 Zeiss microscope (Jena, Germany) was used for the fluorescence assay and samples imaging. Remaining cells were collected, fixed in 70 wt% ethanol overnight at −20 °C and washed twice with PBS. Then, the cells were incubated with 100 μg/mL PI for 30 min and shielded against light at room temperature before flow cytometric analysis. DNA content and cell cycle distribution were monitored using a XML Beckman Coulter cytometer and measured using FlowJo software (Indianapolis, IN, USA).

## 3. Results and Discussion

### 3.1. Coll/Zn^2+^-CaPs Biocomposite Coatings: Structural and Morphological Characterization

#### 3.1.1. FTIR

**Figure 2:**
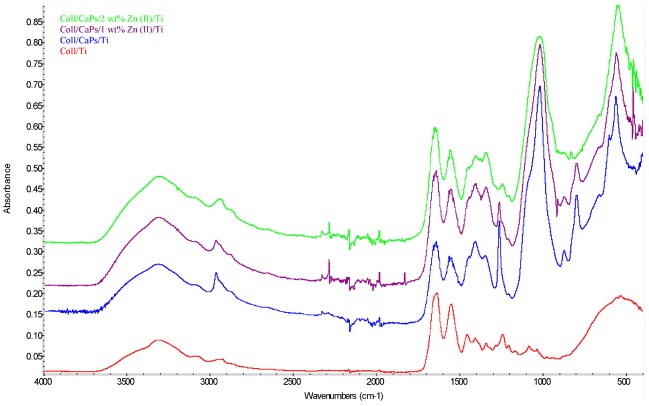
FTIR spectra of the Coll/Zn(II)-CPs biocomposites deposited on Ti substrates.

Structural modifications in the collagen-calcium phosphate association were monitored by FTIR analysis (Figure 2). The use of one or another of the methods for thin films deposition did not significantly influenced the functional changes in the hybrid assembly (at 1032 cm^−1^ specific to phosphate units and 1242 cm^−1^ specific to amide collagen bonds).

The Zn^2+^ content stands for a key factor in determining the molecular synergism between the collagen and inorganic network. An increase of the Zn^2+^ amount induces a gradual decrease of the absorbance in phosphate and amide regions. A large substitution of Ca^2+^ by Zn^2+^ generates structural modifications of apatite pattern, in particular for phosphate groups. These permutations of the P-O and/or P–OH functions stay at the origin of weak interactions and complementarity functional effects between the organic biopolymer (e.g., –OH, –COOH, –NH_2_ groups of collagen) and phosphate moieties in Zn^2+^-substituted CaPs.

#### 3.1.2. IR Microscopy

**Figure 3:**
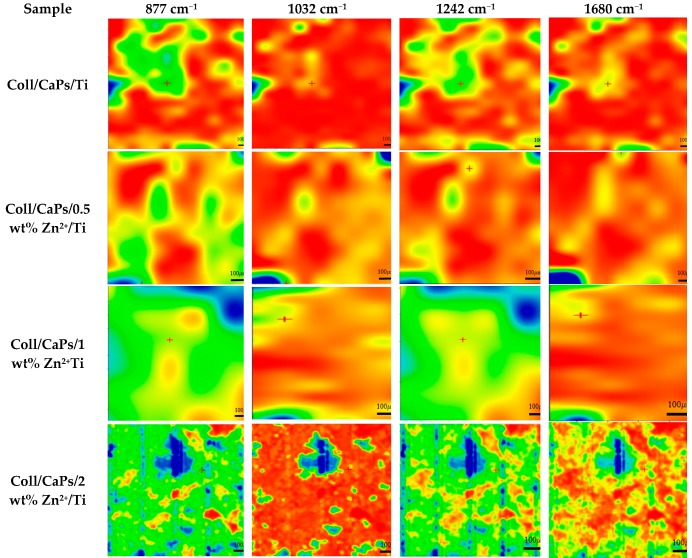
IR mapping of Coll/Zn(II)-CPs thin films deposited on Ti substrate by spin coating.

The high homogeneity of thin films deposited on biocompatible Ti substrates is a mandatory condition in the development of an implant prototype with enhanced biological behavior [28]. IR mapping analysis of the biocomposite films deposited by spin coating is illustrated in Figure 3.

Direct CaPs formation in the collagen matrix can be monitored based on the peak at 1032 cm^−1^. On the other side, the formation of carbonated apatite can be followed based on the peak at 877 cm^−1^. The organic phase can be assigned according to the bands peaking at 1680 and 1242 cm^−1^. In fact, the band at 1242 cm^−1^ can be also used to evaluate the collagen integrity in depositions. The decreases in intensity of the highest Zn^2+^ fraction peaking at 1032 cm^−1^, can be accounted by cation substitutions in the apatite network. Accordingly, an excess in the Zn^2+^ concentration results in an enhanced saturation of apatite units. More Ca^2+^ ions are replaced by Zn^2+^, promoting smooth diffused areas in both phosphate region and amide section [29].

The analysis of the maps recorded for the samples deposited by spin coating (Figure 3) showed a non-uniform layer even when using collagen. In the case of composite hydrogel depositions on Ti, large agglomerates could be observed onto the surface of the metallic substrate. These conglomerates can reach hundreds of microns (~500 µm) as clearly visible for Coll/2 wt% Zn^2+^-CaPs sample.

**Figure 4:**
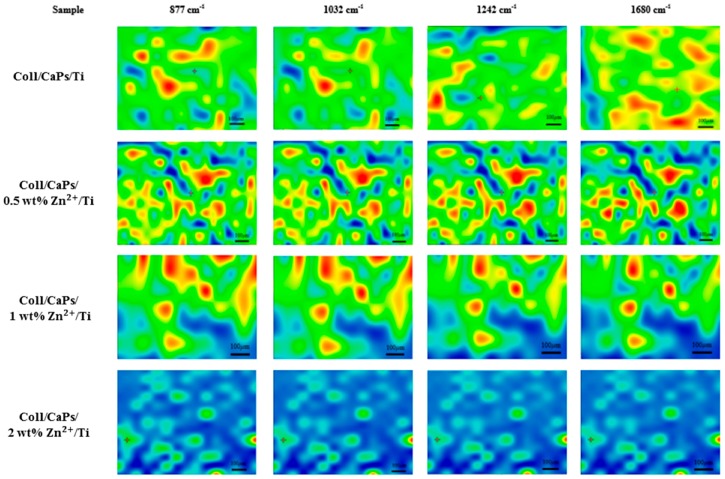
IR mapping of Coll/Zn(II)-CPs thin films deposited on Ti substrate by MAPLE.

An essential remark in case of MAPLE films (Figure 4) is the advanced uniformity of the composite mixture on Ti substrates.

A significant modification is visible in the amide region (1680–1242 cm^−1^) of the Coll/CaPs composite that is more uniform than for the film deposited by spin coating. One may consider this feature due to the structural conservation of the collagen molecule in terms of thermal denaturation. The cleavage of peptide bonds in the collagen structure is restricted because of the low temperature during the MAPLE deposition process, while thin film quality is significantly improved. In fact, even the maps recorded for all of the four wavenumbers are quite similar, there are only marginal differences which confirm that neither compositional nor morphological (thickness) heterogeneities are present.

#### 3.1.3. SEM

**Figure 5:**
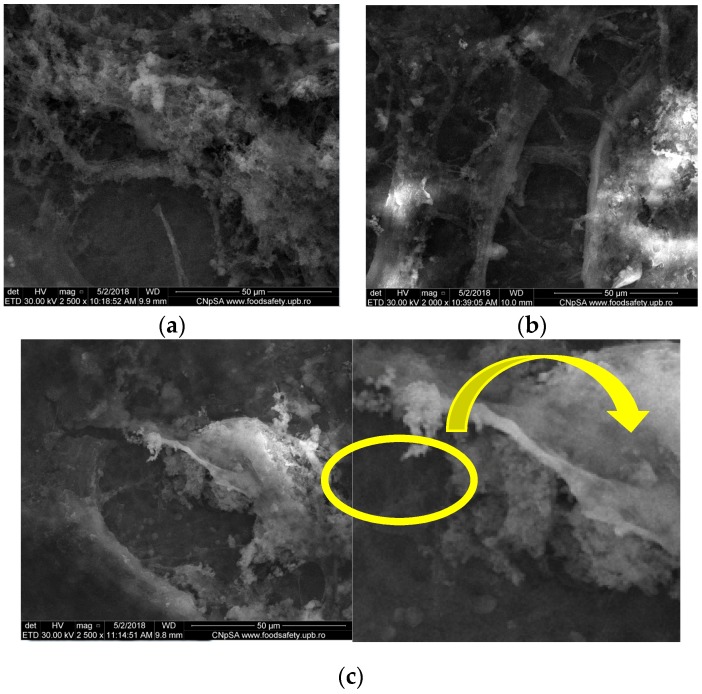
Scanning electron microscopy (SEM) images of Coll/CaPs thin films deposited by spin coating with different Zn(II) fractions (**a**) 0 wt%; (**b**) 1 wt%; (**c**) 2 wt%.

SEM images of spin coating deposited films (Figure 5) suggest that collagen is maintaining its native fibrillary structure. Although collagen and CaPs are intimately covering Ti, highly rough surfaces are obtained. A high Zn^2+^ content (2 wt%) results in a distinct morphology, characterized by small and thin particles growing inside the organic matrix (Figure 5c, yellow area). One may therefore assume that a Zn^2+^ content higher than 1 wt% could promote the crystallization process of calcium phosphates. This specific feature is significant for the development of artificial osseous regeneration [30].

**Figure 6:**
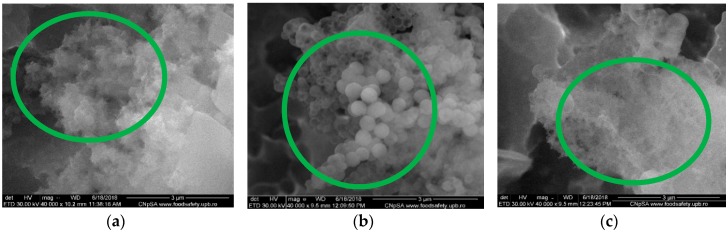
SEM images of the Coll/CaPs thin films deposited by MAPLE with different Zn(II) content (**a**) 0 wt%; (**b**) 1 wt%; (**c**) 2 wt%.

The preservation of the collagen structural pattern after MAPLE deposition has improved the homogeneity and morphology of the thin films (Figure 6). It should be emphasized that a highly ordered organic matrix that accommodates the inorganic particles has been formed, anticipating a potential osteo-integration behavior of the coatings. As observed, a closely-packed collagen-like architecture promotes an advanced crystallization of the substituted CaPs, proportional with the Zn^2+^ amount increase, similar to films deposited by spin coating. The difference in case of MAPLE-deposition method resides in the morphology of the particles developing inside the collagen pattern. The formation of diffused agglomerations associated to HAp was noticed for un-substituted Coll/CaPs (Figure 6a). Furthermore, different spherical nano-structures similar to CaP morphology were observed (Figure 6b,c). Small clusters were evidenced for up to 1 wt% Zn^2+^ (Figure 6c). The size decrease of droplets could be attributed to a possible Zn^2+^ super-saturation of the apatite network. The supplementary Zn-O bonds are placed outside calcium phosphate particles [31].

### 3.2. In-Vitro SBF Assay of Coll/Zn^2+^-CaPs Biocomposite Coatings

#### 3.2.1. FTIR

**Figure 7:**
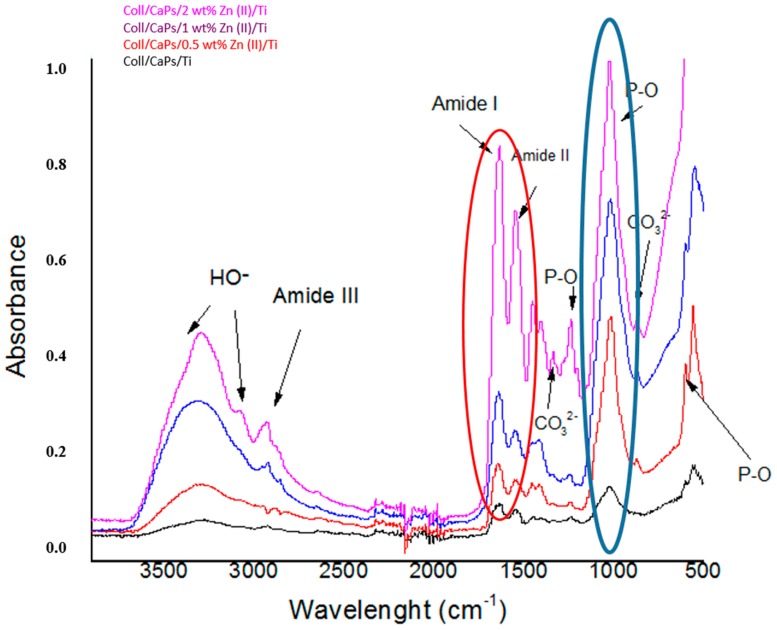
FTIR spectra of the Coll/Zn(II)-CPs thin films deposited by spin coating after 14 days of immersion in SBF.

FTIR recorded spectra were different before (Section 3.1.1) and after immersion in SBF (Figure 7), in particular in the phosphate and amide regions, probably due to the in-vitro influence of the Zn^2+^ substitutional agent.

The increase in Zn^2+^ induces a rise of the P-O band absorbance due to the enhanced formation of HAp, as a result of ionic exchanges during the immersion in SBF. In this context, possible cationic migrations in interstitial positions could be envisaged, followed by the generation of new phosphate functions able to bind positively charged species or interact with collagen moieties. In addition, the preservation of the inorganic template is respected to assure an adequate confinement with the collagen matrix (see the amide regions in red circle in Figure 7). The same behavior was expected for the MAPLE-deposited coatings: a decrease in the intensity of the amide and phosphate bands in respect with spin coating depositions which could be assigned to a very thin layer formation on the Ti substrate (*Data not shown*).

#### 3.2.2. SEM and EDAX

**Figure 8:**
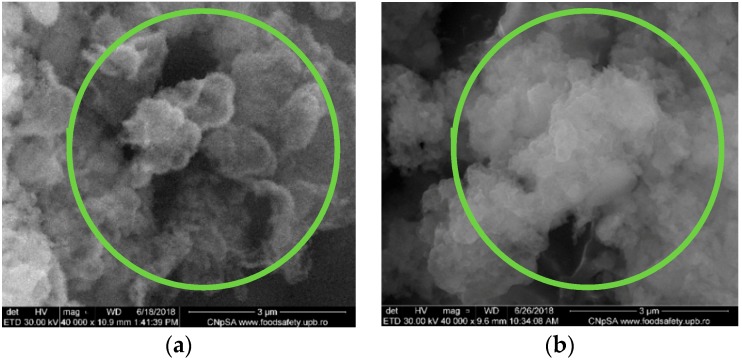
SEM images of the Coll/CaPs thin films with 1 wt% Zn(II)deposited by (**a**) Spin coating; (**b**) MAPLE after 14 days of immersion in SBF.

By correlation with SEM results described in Section 3.1.3, the effect of the immersion in SBF reflected specific modifications in the case of 1wt% Zn^2+^ for both deposition methods. Figure 8 shows the formation of spherical and diffused agglomerations associated to the mineralization process inside the organic template. The further formation of CaPs as a cluster motif is observed in case of MAPLE depositions (Figure 8b, green regions). The collagen structure is not thermally affected in MAPLE, so the definite synergism between the organic network and inorganic particles promotes the HAp crystallization.

**Figure 9:**
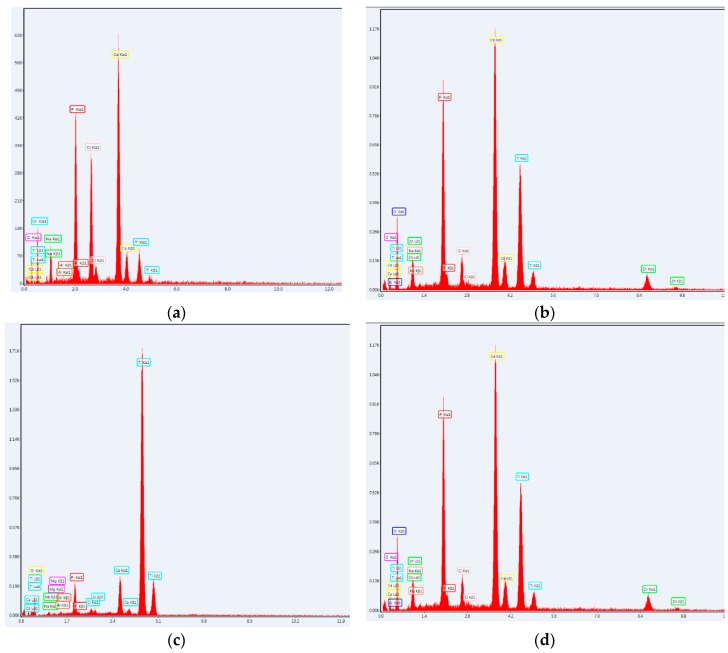
Energy dispersive spectrometer (EDAX), spectra of the Coll/CaPs thin films with 0 wt% and 1 wt% Zn(II) deposited by (**a**,**b**) Spin coating and (**c**,**d**) MAPLE after 14 days of immersion in SBF.

The presence of Zn along with Na, K and Mg, characteristic to the artificial mineralization process, is confirmed by EDAX spectra in Figure 9.

### 3.3. Cytological Assay of Coll/Zn^2+^-CaPs Biocomposite Coatings

**Figure 10:**
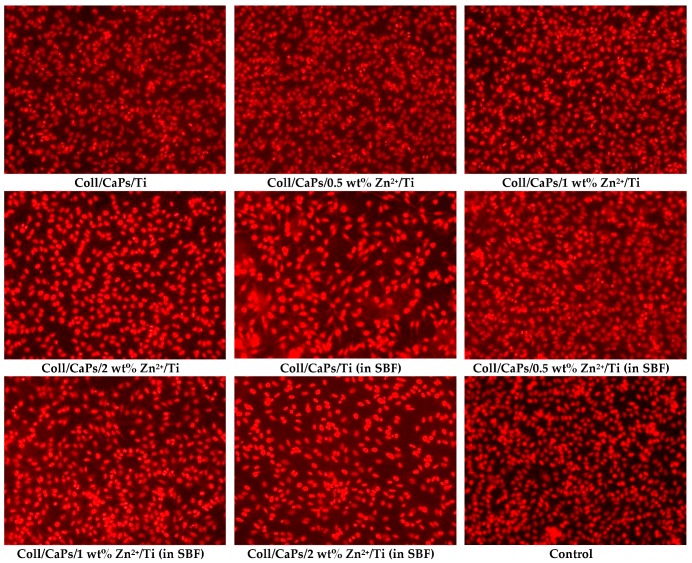
Fluorescent microscopy images of cells grown on Coll/Zn(II)-CPs thin films deposited on Ti by MAPLE.

To determine the usefulness of these materials as medical devices, we conducted a pilot test for cytotoxicity using epithelial cells and a direct contact method that is able to detect weak cytotoxicity. Florescence microscopy revealed that after 24 h of incubation, epithelial cells grown on modified bioactive surfaces present a normal morphology. All tests have been made on the composites deposited by the MAPLE technique. The Coll/Zn^2+^-CaPs biocomposite coatings favored the attachment of cells as visible from Figure 10. The density of the viable cells seems to be the same as for control and normal for this incubation time.

**Figure 11:**
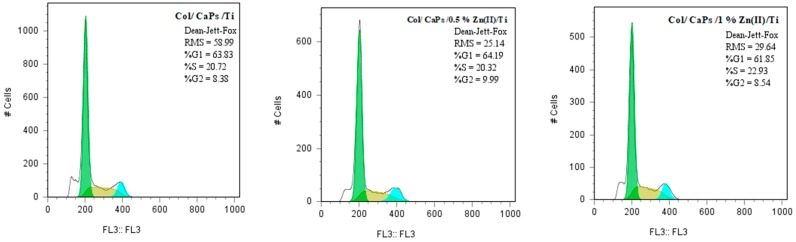

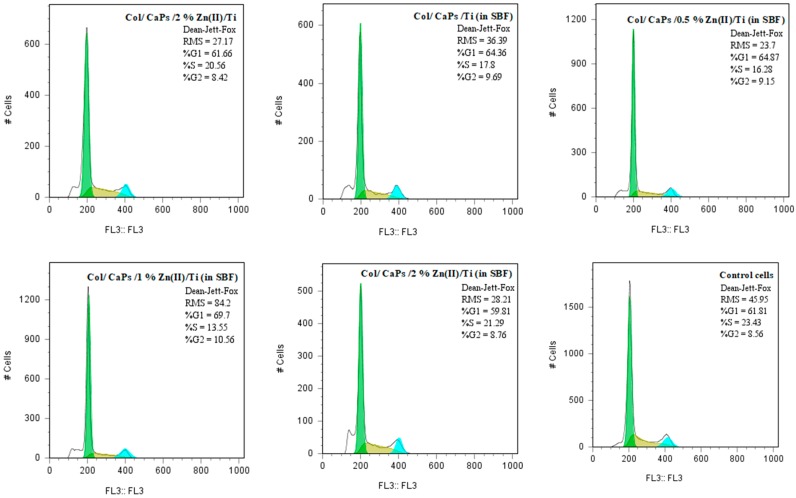
Cell cycle distribution of cells grown on Coll/Zn(II)-CPs thin films deposited on Ti by MAPLE.

Cell cycle analysis (Figure 11) revealed that the compounds immersed in SBF have induced a slight decrease in S phase (DNA replication).

The layers obtained via spin coating were not uniformly distributed on the metallic substrate, as demonstrated by IR microscopy. The heterogeneous deposition of the Coll/Zn^2+^-CaPs biocomposites on Ti substrates generated differences in the biological properties of the implant and therefore no reproducible biological response was obtained and thus not presented.

## 4. Conclusions

A comparative study of spin coating vs. MAPLE depositions for the synthesis of Coll/Zn^2+^-CaPs biocomposite coatings onto chemically etched Ti substrates was carried out. FTIR microscopy demonstrated that the surface homogeneity is superior in case of MAPLE coatings. In both cases, a fraction of 2 wt% Zn^2+^ induced a significant decrease of the absorbance in the P-O and amide regions. However, a further substitution of Ca^2+^ ions is conducting to a decrease of the bands intensity, most probably due to supersaturation. A similar trend in the amide area involves the confinement and synergic effects via weak interactions between the collagen matrix and CaPs particles. Zn^2+^ promoted the in-situ formation of calcium phosphate in collagen matrix, as proved by the presence of spherical CaPs. An excess of Zn^2+^ (up to 1 wt%) generated an uncontrolled crystallization of supplementary Zn–O groups attached to the Zn^2+^-CaPs, in a cluster-like morphology. The in-vitro SBF assay confirmed the gradual formation of supplementary apatite layers in the organic matrix. High density areas were observed in the phosphate region as a result of Ca^2+^-coordinated ions, in conjunction with an increase in the P-O band absorbance. Even in high concentrations, Zn^2+^ promoted the mineralization process, as suggested by an absorbance rise of the P–O band. The SEM analysis of coatings immersed in SBF revealed the effective mineralization of CaPs on MAPLE layers. Well-defined spherical particulates embedded into the collagen matrix proved the growth of a new apatite layer. The collagen structure was preserved under MAPLE, supporting the improved mineralization by weak interactions with numerous active sites. The MAPLE method did not produce secondary compounds that alter cell viability (morphological quantification) or normal cell cycle phases (flow cytometry assessment). One may conclude that Coll/Zn^2+^-CaPs biocomposites synthesized by one-step MAPLE are appropriate for the development of a new generation of surface coated Ti implants with promising biological performances, and encourage us for further testing to demonstrate their capacity to sustain osteoblastic function and bone formation.

## Figures and Tables

**Table 1:** Concentrations of the ionic species used in simulated body fluids (SBF) preparation.

Ionic Species	Na^+^	K^+^	Mg^2+^	Ca^2+^	Cl^−^	HCO_3_^−^	HPO_4_^2−^	SO_4_^2−^
Concentration of the ionic species (mmol/L)	142.0	5.0	1.5	2.5	148.8	4.2	1.0	0.5
